# Clinical Profile of Outpatients Undergoing Swallowing Evaluation and Its Association with Pneumonia History: A Videofluoroscopic Study

**DOI:** 10.3390/jcm15082990

**Published:** 2026-04-15

**Authors:** Ayşe Kübra Söyler, Numan Demir, Selen Serel Arslan, Inci Nur Saltik-Temizel, Ömer Faruk Yaşaroğlu, Hasan Erkan Kilinç, Aynur Ayşe Karaduman

**Affiliations:** 1 Department of Physiotherapy and Rehabilitation, Faculty of Health Science, Aydın Adnan Menderes University, Aydın 09010, Türkiye; ayse.kubra.sahan@adu.edu.tr; 2 Faculty of Physical Therapy and Rehabilitation, Hacettepe University, Ankara 06100, Türkiye; numan@hacettepe.edu.tr (N.D.);; 3 Department of Pediatric Gastroenterology, Hacettepe University Faculty of Medicine, Ankara 06100, Türkiye; 4 Department of Physiotherapy and Rehabilitation, Faculty of Health Science, Harran University, Şanlıurfa 63300, Türkiye; farukyasaroglu@harran.edu.tr; 5 Department of Physiotherapy and Rehabilitation, Faculty of Health Sciences, Lokman Hekim University, Ankara 06890, Türkiye

**Keywords:** dysphagia, silent aspiration, pneumonia, videofluoroscopy

## Abstract

**Background/Objectives**: Dysphagia leads to severe complications, but data on patient profiles are limited in Türkiye. In this study, we present swallowing characteristics across etiologies and examine the association between a history of pneumonia and videofluoroscopic swallowing study (VFSS) findings. **Methods**: This retrospective study included 1055 adults, comprising a large, heterogeneous outpatient population, referred for swallowing evaluation between 2015 and 2018. Clinical data (demographics, diagnoses, feeding status, etc.) and VFSS findings (Penetration–Aspiration Scale, PAS; pharyngeal residue) for liquid and semisolid consistencies were recorded from our electronic database. Associations were initially assessed using the chi-square test. A logistic regression analysis was performed to identify factors associated with pneumonia history. **Results**: Neurologic diseases (62.1%) were the most frequent diagnosis. VFSS identified liquid aspiration in 53.9% (61.8% silent) and semisolid aspiration in 20.1% (72.5% silent) of patients. Pharyngeal residue occurred in up to 10.6% of patients for liquids and 24.3% for semisolids. Diet recommendations were modified for 43.3% of patients (*p* < 0.05). Overall, 26.6% of patients had a history of pneumonia. In unadjusted, exploratory comparisons, pneumonia history was significantly associated with higher frequencies of aspiration and silent aspiration (*p* < 0.001), while pharyngeal residue was comparable between groups (*p* > 0.05). Multivariable logistic regression analysis showed that nonsilent aspiration (PAS 6–7) and silent aspiration (PAS 8) were associated with pneumonia history in both liquid (OR = 2.18 and 3.06) and semisolid consistencies (OR = 2.32 and 3.71), and that pharyngeal residue in semisolid consistency was also associated with pneumonia history (OR = 1.57). **Conclusions**: This study provides a comprehensive clinical profile of dysphagia in Türkiye, highlighting high rates of silent aspiration and the role of instrumental assessment in guiding safe feeding. While a significant association was observed between pneumonia history and impaired swallowing safety and efficiency, the retrospective nature of this study precludes causal interpretations.

## 1. Introduction

Dysphagia is a serious condition that can cause many complications, such as dehydration, malnutrition, aspiration pneumonia, decreased quality of life, and, in some cases, death [1,2,3]. Dysphagia is associated with multiple factors, including aging, comorbidities, and medication use [2]. One of the most important factors affecting the prevalence of dysphagia is etiology [4]. Neurological, psychiatric, cardiopulmonary, and gastroesophageal diseases, structural anomalies, and many types of cancer are among the most common conditions causing dysphagia [3]. The incidence and prevalence of dysphagia are reported to be increasing among adults, with a particularly high risk observed in individuals with stroke, neurodegenerative disease, cancer, and chronic obstructive pulmonary disease [5]. This increases the clinical importance of better characterizing the clinical features of dysphagia across different patient populations. Studies describing patient profiles in dysphagia populations enable us to identify patient characteristics and present experiences from different centers. Although the literature includes profile studies conducted in various countries and clinical settings describing the etiological and clinical characteristics of patients presenting with dysphagia, most have focused on specific diagnostic groups or limited sample sizes and have not comprehensively presented both clinical and instrumental findings; moreover, no comprehensive study defining patient profiles in dysphagia populations has been performed in Türkiye [6,7,8,9,10]. Furthermore, many available profile studies have been conducted within specific clinical settings or disease-oriented cohorts, such as gastroenterology, otorhinolaryngology, or inpatient populations, which may reflect referral patterns and local clinical practices rather than the overall dysphagia population. Gastroenterology-based cohorts frequently report structural and reflux-related esophageal disorders and malignancies, whereas otorhinolaryngology settings more commonly highlight upper aerodigestive tract pathologies [6,8,9]. Consequently, studies presenting clinical and instrumental swallowing findings together in broad and heterogeneous outpatient populations remain relatively limited. The Hacettepe University Swallowing Disorders Research and Application Center is one of the first specialized dysphagia centers in Türkiye, providing multidisciplinary assessment and accepting nationwide referrals. This structure allows us to evaluate a large and heterogeneous patient population with dysphagia. In this context, the patient profile of our department may contribute to a better understanding of dysphagia characteristics in Türkiye, while also providing data that may complement the international literature by offering insights from a large outpatient cohort evaluated in a multidisciplinary referral center.

On the other hand, previous studies have reported that swallowing-related abnormalities identified during instrumental swallowing assessment, including aspiration, silent aspiration, and pharyngeal residue, are more frequently observed in patients with pneumonia [11,12,13,14,15]. In addition, VFSS-based investigations have shown that patients with pneumonia present with higher Penetration–Aspiration Scale (PAS) scores and greater amounts of pharyngeal residue compared with those without pneumonia, while silent aspiration has been reported to show a stronger association with pneumonia than penetration or aspiration [11,14]. The videofluoroscopic swallowing study (VFSS), which is considered the gold standard for the assessment of swallowing function, enables the objective evaluation of these swallowing abnormalities [2]. However, studies evaluating key VFSS findings and comparing patients with and without a history of pneumonia in broad patient populations remain limited. Therefore, examining the association between VFSS findings and pneumonia history may contribute to a better understanding of the swallowing characteristics observed in this patient population.

Accordingly, the primary aim of this study was to describe dysphagia characteristics using clinical and instrumental assessments in adult outpatients referred for VFSS. The secondary aim was to examine the association between a history of pneumonia and the presence of nonsilent aspiration, silent aspiration, and pharyngeal residue.

## 2. Materials and Methods

### 2.1. Study Design and Participants

The study protocol was approved by the Hacettepe University Non-Interventional Clinical Research Ethics Committee (approval number: GO 19/436, date: 24 April 2019). In this single-center study, the records of patients admitted to Hacettepe University Swallowing Disorders Research and Application Center with suspicion of dysphagia between 2015 and 2018 were reviewed retrospectively. The inclusion criteria were the following: (1) age ≥18 years, (2) being evaluated as an outpatient, (3) referral to the center with suspicion of dysphagia during the study period, and (4) completion of both clinical swallowing evaluation and VFSS. The exclusion criterion was the presence of missing or incomplete clinical or VFSS data required for study analyses. When multiple clinical swallowing evaluations or VFSS examinations were available for the same patient, only the initial assessment was included in the analysis.

### 2.2. Variables

Data were retrieved from the clinical swallowing evaluation and VFSS records available in the electronic database. From clinical swallowing evaluation records, demographic characteristics (age, height, weight, body mass index, and gender), medical diagnoses, Eating Assessment Tool (EAT-10) scores, feeding type before swallowing evaluation, and recommended feeding type after swallowing evaluation were extracted. From VFSS records, Penetration–Aspiration Scale (PAS) scores for liquid and semisolid consistencies and the presence of pharyngeal residue in the valleculae and pyriform sinuses were recorded for analysis. In addition, information regarding pneumonia history was identified retrospectively based on documented physician diagnoses recorded in the hospital electronic medical records. These records included the physician notes and consultation reports available in the patient files.

The Turkish version of the Eating Assessment Tool-10 (EAT-10) was used as part of the routine clinical swallowing assessment in our department to determine dysphagia symptom severity. The assessment tool, which is frequently used in dysphagia assessment, is a self-administered questionnaire consisting of 10 items, each scored from 0 (no problems) to 4 (severe problems), yielding a total score ranging from 0 to 40, with higher scores indicating greater swallowing-related symptom severity [16]. As part of our routine clinical practice, the questionnaire is explained to patients, and they are asked to complete it independently. Assistance is provided when necessary for patients who have difficulty completing the questionnaire, without influencing their responses. VFSS was used for the instrumental evaluation of dysphagia in our department and was conducted in accordance with the technique recommended by Logemann [17]. During the examination, patients were seated upright, and swallowing was recorded in the lateral plane at a rate of 30 frames per second. The oral and pharyngeal phases of swallowing were evaluated using a thin liquid (water) and semisolid (yogurt). According to our institutional VFSS protocol, each bolus consistency is generally administered for up to three swallowing trials, with an average bolus volume of approximately 3 mL [18]. However, the exact number of trials per consistency was not fixed and could vary depending on the patient’s known swallowing function based on prior clinical assessment and the aspiration risk observed during VFSS. The examination could be terminated at the discretion of the evaluator if an increased risk of aspiration was identified to ensure patient safety. The Penetration–Aspiration Scale (PAS) was used to assess airway invasion during VFSS. When multiple swallowing trials were performed for the same consistency, the maximum (worst) PAS score observed for each patient was used for analysis. PAS scores were analyzed on a per-patient basis rather than per individual bolus. According to the 8-point PAS, a score of 1 indicates no airway invasion, scores of 2–5 indicate penetration, and scores of 6–8 indicate airway aspiration. Also, a score of 8 indicates aspiration in which material enters the airway with no attempt to clear and is defined as silent aspiration [19]. In addition, pharyngeal residue in valleculae and pyriform sinuses was recorded as “present” or “absent” for both liquid and semisolid consistencies [18].

Patients were classified according to their diagnosis as having neurologic diseases, cardiac diseases, cancer, structural anomalies, metabolic diseases, gastrointestinal diseases, and psychiatric diseases. Patients who were not included in one of these groups were evaluated as “others”. Patients who did not have a diagnosis that could cause dysphagia were evaluated as a separate group (without any diagnosis).

To describe patient profiles, demographic characteristics, EAT-10 scores, VFSS findings (PAS scores and residue), feeding status before and after swallowing evaluation, and pneumonia history were descriptively summarized across etiological groups. In addition, patients were grouped according to the presence or absence of pneumonia history. Aspiration patterns were categorized based on PAS scores as no aspiration (PAS 1–5), nonsilent aspiration (PAS 6–7), and silent aspiration (PAS 8). Pharyngeal residue (present/absent) for liquid and semisolid consistencies was also defined for further analyses.

### 2.3. Statistical Analysis

Statistical analyses were performed using the IBM SPSS Statistics for Windows, Version 26.0 (IBM Corp., Armonk, NY, USA). Descriptive analyses were calculated as mean ± standard deviation for quantitative data, and number/percent (*n*/%) for qualitative data. The change between the feeding type at baseline (before swallowing evaluation) and the recommended feeding type according to swallowing evaluation was compared using the McNemar test. Patients were grouped according to the presence of a history of pneumonia. The association between a history of pneumonia and aspiration patterns (no aspiration, nonsilent aspiration, and silent aspiration) and pharyngeal residue was initially examined using the chi-square test, with pneumonia history (yes/no) defined as the dependent variable. A logistic regression analysis was performed to identify factors associated with pneumonia history. Aspiration patterns were entered as categorical variables, with “no aspiration” as the reference category. Pharyngeal residue for liquid and semisolid consistencies was included as an independent variable in the model. Odds ratios (ORs) with 95% confidence intervals (CIs) were calculated. A *p*-value < 0.05 was considered statistically significant.

## 3. Results

A total of 1055 adult outpatients were included in the analysis, and their demographic characteristics and etiological subgroups are shown in Table 1. The most common diagnosis of patients who were referred to our department was neurologic diseases (62.1%), followed by cardiac diseases (8.9%), and cancer (7.7%). Additionally, 5.1% of patients referred with suspected dysphagia did not have an identified medical diagnosis. The mean EAT-10 score of all patients was 17.41 ± 11.35 (subgroups ranged between 15.13 ± 10.77 and 21.24 ± 14.09), with higher scores observed in patients with cancer. The PAS scores and the presence of pharyngeal residue are presented in Table 2. The mean PAS score was 4.82 ± 3.14 for liquids and 2.56 ± 2.70 for semisolids. Based on the PAS frequency distribution, aspiration was observed in 53.9% of patients during liquid swallowing and in 20.1% during semisolid swallowing. Among those with aspiration, silent aspiration accounted for 61.8% of liquid aspirations and 72.5% of semisolid aspirations. Vallecular residue was observed in 10.6% of patients for liquid and 24.4% for semisolid consistencies, whereas pyriform sinus residue was present in 8.9% of patients for liquids and 18.7% for semisolid consistencies. Diet recommendations were changed for 43.3% of all patients following swallowing evaluation. There was a significant difference between feeding types before and after swallowing evaluation for all patients and all subgroups except other diagnoses (*p* < 0.05). Feeding types before and after swallowing evaluation are presented in Table 3. Before swallowing evaluation, 65.5% of patients were receiving an oral diet and 3.4% were on a liquid-restricted diet. Following swallowing evaluation, the proportion of patients on an oral diet decreased to 35.1%, while 32.9% of patients were recommended a liquid-restricted diet (Table 3). Overall, 26.6% of the patients had a history of pneumonia, with the highest proportion observed in the cardiac disease group (51.2%) (Table 3). In unadjusted comparisons, patients with a history of pneumonia demonstrated higher frequencies of aspiration and silent aspiration in VFSS for both liquid and semisolid consistencies compared with those without a history of pneumonia (*p* < 0.001). In contrast, the presence of pharyngeal residue did not differ significantly between patients with and without a history of pneumonia for either consistency (*p* > 0.05) (Table 4). Multivariable logistic regression analysis showed that nonsilent aspiration (PAS 6–7) was associated with pneumonia history for liquid (OR = 2.18, 95% CI: 1.26–3.78, *p* = 0.005) and semisolid consistencies (OR = 2.32, 95% CI: 1.32–4.08, *p* = 0.003), while silent aspiration (PAS 8) was associated with pneumonia history for liquid (OR = 3.06, 95% CI: 1.98–4.72, *p* < 0.001) and semisolid consistencies (OR = 3.71, 95% CI: 2.15–6.40, *p* < 0.001). Pharyngeal residue for semisolid consistency was found to be significantly associated with pneumonia history in the multivariable model (OR = 1.57, 95% CI: 1.09–2.26, *p* = 0.015) (Table 5).

## 4. Discussion

This large, structured study, including 1055 adult outpatients with suspected dysphagia, presents the overall dysphagia profile of a tertiary referral center in Türkiye. In this study, patients’ swallowing safety and efficiency were evaluated using both clinical swallowing examinations and the VFSS. Unadjusted comparisons showed that impaired swallowing safety parameters, including aspiration and silent aspiration, were observed across both liquid and semisolid bolus consistencies more frequently in patients with a history of pneumonia than in those without such a history. Furthermore, multivariable logistic regression analysis demonstrated that both nonsilent and silent aspiration were independently associated with pneumonia history, with silent aspiration showing a stronger association across consistencies. Additionally, pharyngeal residue for semisolid consistencies was identified as an independent factor associated with pneumonia history.

Overall, the patients had an average age of 59.74 ± 18.90 years, and 58% were male. It was determined that the male/female ratio of the patients was 1.38:1. It was also observed that patients referred with suspicion of dysphagia presented with a wide range of underlying clinical conditions, including neurologic, cardiac, and psychiatric disorders, with neurologic diseases representing the most frequent diagnostic category. These findings reflect the heterogeneous clinical profile of patients referred for dysphagia evaluation in this tertiary referral center. Additionally, 5.1% of the patients referred with suspected dysphagia had no identified medical diagnosis. Dysphagia-related complaints may also be observed in association with aging and psychological factors [20,21]. The mean age of patients in this subgroup was 54.09 ± 18.54 years, and age-related physiological changes in swallowing may also be related to these complaints [20].

It was determined that two-thirds of the patients had impaired swallowing safety for liquid consistencies, characterized by penetration or aspiration, and approximately one-third of the patients had impaired swallowing safety for semisolid consistencies. Although dysphagia was common in our sample, fortunately, most patients were suitable for a liquid-restricted modified oral intake. An important finding from our study is that the silent aspiration rate is quite high for both consistencies. In a study that evaluated 2000 patients using VFSS, silent aspiration was reported in 55% of patients who aspirated [22]. Another study evaluated 455 patients using FEES and reported that silent aspiration ranged between 11.7% and 50% depending on the etiology [23]. Within this context, the frequency of silent aspiration observed in our sample appears to be higher than the ranges reported in previous studies. Silent aspiration is a significant problem in many respects. When foreign material is aspirated into the airway, it may descend into the tracheobronchial tree due to the absence of a protective cough reflex [22,23]. This may lead to serious complications such as aspiration pneumonia [22]. Additionally, the absence of signs of aspiration in patients with silent aspiration reduces the awareness of the patient, family, and clinician about aspiration [22], and it has been reported that up to 50% of silent aspiration may be overlooked in clinical swallowing evaluation [23]. Therefore, these patients may be more vulnerable to pulmonary complications, since silent aspiration allows foreign material to be exposed to the lungs for a longer period of time [22]. This result demonstrates the importance of instrumental swallowing evaluation in patients suspected of having silent aspiration. Although comparatively less frequent for liquids, it was determined that up to one-quarter of the patients had an impaired swallowing efficiency for semisolid consistencies, as indicated by the presence of pharyngeal residue. In a VFSS including 305 adults evaluated for suspected dysphagia, pharyngeal residue was reported in approximately 37% of all analyzed boluses across different consistencies (thin, 36%; mildly thick, 41%; moderately thick, 35%) [24]. In addition, consistent with previous study results, vallecular residue was more frequent than pyriform sinus residue across both consistencies [24,25]. Although there is no consensus on the more dangerous location of pharyngeal residue, some studies have reported that residue located in the piriform sinuses is associated with a higher risk of aspiration than residue in the vallecula in terms of swallowing safety [26,27]. In this context, evaluating the presence and anatomical location of pharyngeal residue may contribute to a more comprehensive assessment of swallowing safety. Following swallowing evaluation, dietary modifications were recommended for approximately half of the patients, most commonly involving a transition from a non-restricted oral diet to a liquid-restricted diet. This finding suggests that, in a proportion of patients, the baseline dietary practices prior to instrumental assessment may not have been fully aligned with swallowing safety. However, dietary changes were reported in a general manner, and food and liquid consistencies were not classified in detail according to the internationally accepted International Dysphagia Diet Standardization Initiative (IDDSI) framework; this limits the extent to which findings related to dietary modifications can be interpreted in detail [28].

In this study, more than one-quarter of patients had a history of pneumonia, and the cardiac disease group had the highest prevalence of pneumonia among all diagnostic categories. Previous studies have reported varying rates of pneumonia among patients with dysphagia, with incidences ranging between 11.7% and 22% in large cohorts [12,15]. Differences in the reported pneumonia rates across studies are likely influenced by variations in study design and patient characteristics. Overall, the prevalence of pneumonia observed in our study appears consistent with the previously reported ranges. In our sample, unadjusted comparisons showed that aspiration and silent aspiration were observed more frequently in patients with a history of pneumonia than in those without such a history, whereas the presence of pharyngeal residue did not differ significantly between the two groups. However, pharyngeal residue was assessed only as a binary variable (present/absent) without a standardized grading of residue severity or clearance, which may have limited the ability to detect more nuanced associations with pneumonia history. Additionally, pharyngeal residue may still contribute to post-swallow aspiration risk, which cannot be detected in the current assessment approach. When these findings were examined in a multivariable model, both nonsilent and silent aspiration remained independently associated with pneumonia history, with consistently stronger associations observed for silent aspiration across consistencies. Notably, silent aspiration was associated with approximately 3-fold higher odds of pneumonia, whereas nonsilent aspiration was associated with approximately 2-fold higher odds. In addition, pharyngeal residue for semisolid consistencies emerged as an independent factor (OR: 1.57). Previous studies have reported aspiration, silent aspiration, and pharyngeal residue as being factors associated with pneumonia, and higher PAS scores are associated with an increased risk of pneumonia [11,12,14]. However, the extent to which these factors are independently associated with pneumonia remains unclear. A large-scale VFSS showed that the risk of pneumonia increased with the severity of airway invasion, with laryngeal penetration, tracheobronchial aspiration, and especially silent aspiration being associated with approximately 4-fold, 10-fold, and 13-fold increased risks, respectively, supporting the clinical significance of aspiration severity [14]. Similarly, Kim et al. demonstrated that silent aspiration was associated with pneumonia, highlighting the importance of impaired protective mechanisms such as the cough reflex [12]. However, not all studies have identified aspiration as an independent predictor of pneumonia. Ko et al. reported that, although aspiration findings and PAS scores were significantly higher in patients with pneumonia, these variables did not maintain their independent significance in multivariable models. Instead, temporal parameters such as pharyngeal delay time and pharyngeal transit time emerged as stronger predictors [11]. This suggests that, although aspiration is associated with pneumonia, it may not independently explain the risk, and that additional physiological impairments in swallowing may contribute to the development of pneumonia. Consistent with the literature, our findings support the multifactorial nature of aspiration pneumonia. The stronger association observed for silent aspiration compared to nonsilent aspiration suggests that impaired airway protective mechanisms may play a significant role. Additionally, the identification of pharyngeal residue for semisolid consistencies as an independent factor indicates that not only aspiration but also impaired bolus clearance and the potential for post-swallow aspiration should be considered. Overall, these results suggest that pneumonia risk is better understood through a comprehensive evaluation of aspiration characteristics, swallowing timing, and bolus clearance-related physiological components.

In the present study, a significant association between key swallowing impairments, including aspiration, silent aspiration, and pharyngeal residue, and a history of pneumonia was observed in a large and heterogeneous population. However, due to the retrospective design of the study and the fact that VFSS assessments were performed after documented pneumonia episodes, causal inferences cannot be established. It remains unclear whether aspiration or pharyngeal residue contributed to the development of pneumonia or whether pneumonia and its related clinical processes subsequently affected swallowing physiology. Moreover, pneumonia is a multifactorial condition influenced by factors such as age, comorbidities, nutritional status, and susceptibility to infection, with dysphagia representing one component of this complex interaction [29]. Future studies with prospective designs that consider these potential factors may help to better clarify the temporal relationship between swallowing safety and efficiency and pneumonia.

The present study provides a broad overview of swallowing safety and efficiency evaluated using VFSS for a large outpatient population at a tertiary referral center. The main limitation of this study is the absence of prospective follow-up after the swallowing evaluation, which prevented the evaluation of subsequent pneumonia development and temporal relationships between VFSS findings and clinical outcomes. In addition, several other limitations of this study should be acknowledged. While patients in this study were classified based on the presence of a history of pneumonia, the specific type of pneumonia could not be differentiated in detail. Therefore, it cannot be assumed that all pneumonia cases represent aspiration pneumonia, and the findings should be interpreted within this context. Moreover, due to the retrospective nature of the study, the bolus consistencies used during VFSS were not standardized according to the internationally accepted IDDSI framework. The lack of routine clinical implementation of IDDSI in our department during the study period precluded the use of a standardized approach for bolus preparation and reporting. This limitation may further restrict detailed comparisons between swallowing evaluation findings and dietary recommendations.

Future studies are recommended to standardize swallowing assessments and dietary status classifications according to the IDDSI framework and to adopt standardized approaches for bolus preparation and reporting to improve comparability across studies. We also recommend the use of validated grading scales to quantify the severity of pharyngeal residue to enable a more detailed evaluation of its potential contribution to swallowing safety and related clinical outcomes. Furthermore, we recommend that future research should differentiate aspiration pneumonia from other types of pneumonia to better clarify the relationship between swallowing impairment and pneumonia development.

## 5. Conclusions

This study provides a comprehensive overview of swallowing characteristics in a large outpatient population referred for dysphagia evaluation in a tertiary center. Neurologic diseases represented the most common underlying diagnosis, and impaired swallowing safety, particularly aspiration and silent aspiration, was frequently observed. Patients with a history of pneumonia demonstrated higher frequencies of aspiration and silent aspiration. Importantly, multivariable analysis identified nonsilent and silent aspiration, particularly silent aspiration, as independent factors associated with pneumonia history, along with pharyngeal residue for semisolid consistencies. These findings highlight the clinical importance of instrumental swallowing assessment, particularly in patients suspected of having swallowing safety impairments or silent aspiration. However, due to the retrospective design and potential confounding factors in this study, causal relationships cannot be established. There is a need for prospective studies that also consider potential confounding and related factors to more clearly elucidate the relationship between dysphagia and pneumonia in this patient population.

## Figures and Tables

**Table 1:** Demographic characteristics of all patients and subgroups according to their etiology.

	Total (*n* = 1055)	Neurologic Diseases (*n* = 655)	Cardiac Diseases (*n* = 94)	Cancer (*n* = 81)	Structural Anomalies (*n* = 42)	Metabolic Diseases (*n* = 38)	Gastrointestinal Diseases (*n* = 38)	Psychiatric Diseases (*n* = 28)	Others (*n* = 24)	Without Any Diagnosis (*n* = 54)
**Gender**										
Male	611 (58%)	390 (59.7%)	63 (67%)	56 (69.1%)	16 (38.1%)	12 (31.6%)	21 (55.3%)	15 (53.6%)	12 (50%)	25 (46.3%)
Female	442 (42%)	263 (40.3%)	31 (33.0%)	25 (30.9%)	26 (61.9%)	26 (68.4%)	17 (44.7%)	13 (46.4%)	12 (50%)	29 (53.7%)
**Mean (SD)**										
**Age (years)**	59.74 ± 18.90	60.84 ± 18.60	68.84 ± 18.25	59.93 ± 16.52	52.90 ± 19.68	55.34 ± 19.14	54.47 ± 17.58	42.92 ± 16.69	53.33 ± 18.17	54.09 ± 18.54
**Height (cm)**	166.32 ± 9.49	166.37 ± 9.38	165.48 ± 9.32	167.66 ± 9.06	165.32 ± 8.30	161.93 ± 9.20	169.21 ± 7.65	168.96 ± 7.51	161.11 ± 14.84	167.04 ± 11.26
**Weight (kg)**	67.47 ± 15.25	68.22 ± 15.06	68.70 ± 17.31	63.87 ± 13.60	60.05 ± 14.98	69.27 ± 17.86	71.30 ± 14.65	63.07 ± 11.55	61.00 ± 17.91	68.97 ± 13.04
**BMI (kg/m^2^)**	24.43 ± 5.27	24.70 ± 5.18	24.85 ± 5.48	22.71 ± 4.46	22.29 ± 5.76	26.50 ± 7.35	24.91 ± 4.82	22.10 ± 3.82	23.03 ± 4.97	24.99 ± 5.05

*n*: number of enrolled patients; SD: standard deviation.

**Table 2:** Videofluoroscopic swallowing study results for patients.

	Total	Neurologic Diseases	Cardiac Diseases	Cancer	Structural Anomalies	Metabolic Diseases	Gastrointestinal Diseases	Psychiatric Diseases	Others	Without Any Diagnosis
**Mean (SD)**										
**PAS score—liquid**	4.82 ± 3.14	5.33 ± 3.00	5.03 ± 3.18	5.33 ± 2.95	3.56 ± 2.99	3.89 ± 3.22	2.41 ± 2.69	3.81 ± 3.29	3.25 ± 2.96	2.29 ± 2.61
**PAS score—semisolid**	2.56 ± 2.70	2.64 ± 2.74	2.75 ± 2.79	3.79 ± 3.10	1.84 ± 2.02	2 ± 2.34	1.38 ± 1.62	2.32 ± 2.63	1.95 ± 2.23	1.70 ± 2.07
**Presence of pharyngeal residue, number (%)**										
**Vallecula—liquid**	112 (10.6)	77 (11.8)	9 (9.6)	12 (14.8)	4 (9.5)	1 (2.7)	2 (5.3)	1 (3.6)	3 (12.5)	3 (5.6)
**Pyriform sinus—liquid**	94 (8.9)	67 (10.2)	9 (9.6)	8 (9.9)	3 (7.1)	0	2 (5.3)	1 (3.6)	2 (8.3)	2 (3.7)
**Vallecula—semisolid**	256 (24.3)	183 (27.9)	23 (24.5)	22 (27.1)	9 (21.4)	3 (7.9)	2 (5.3)	1 (3.6)	6 (25.0)	7 (13.0)
**Pyriform sinus—semisolid**	197 (18.7)	141 (21.5)	19 (20.2)	14 (17.3)	6 (14.3)	1 (2.6)	3 (7.9)	2 (7.1)	5 (20.8)	6 (11.1)

PAS: Penetration–Aspiration Scale; 1 = no airway invasion; 2–5 = penetration; 6–7 = nonsilent aspiration; 8 = silent aspiration.

**Table 3:** Pneumonia history and feeding type before and after swallowing evaluation.

	Total	Neurologic Diseases	Cardiac Diseases	Cancer	Structural Anomalies	Metabolic Diseases	Gastrointestinal Diseases	Psychiatric Diseases	Others	Without Any Diagnosis
**Feeding type before swallowing evaluation, number (%)**										
**Oral**	642 (65.5)	377 (61.7)	52 (65.0)	34 (46.6)	33 (84.6)	27 (77.1)	33 (89.2)	19 (70.4)	14 (58.3)	52 (98.1)
**Liquid-restricted**	33 (3.4)	27 (4.4)	1 (1.3)	1 (1.4)	2 (5.1)	1 (2.9)	0	0	1 (4.2)	0
**Non-oral**	305 (31.1)	207 (33.9)	27 (33.8)	38 (52.1)	4 (10.3)	7 (20.0)	4 (10.8)	8 (29.6)	9 (37.5)	1 (1.9)
**Recommended feeding type after swallowing evaluation, number (%)**										
**Oral**	334 (35.0)	153 (25.6)	30 (34.5)	23 (31.1)	17 (54.8)	17 (48.6)	23 (74.2)	14 (56.0)	14 (63.6)	43 (82.7)
**Liquid-restricted**	314 (32.9)	241 (40.4)	25 (28.7)	13 (17.6)	10 (32.3)	10 (28.6)	6 (19.4)	3 (12.0)	2 (9.1)	4 (7.7)
**Non-oral**	306 (32.1)	203 (34.0)	32 (36.8)	38 (51.4)	4 (12.9)	8 (22.9)	2 (6.5)	8 (32.0)	6 (27.3)	5 (9.6)
**Pneumonia history, *n* (%)**										
**No**	774 (73.4%)	481 (73.4%)	46 (48.9%)	63 (77.6%)	32 (76.9%)	31 (81.6%)	35 (90.9%)	19 (66.7%)	17 (71.4%)	50 (92.6%)
**Yes**	281(26.6%)	174 (26.6%)	48 (51.1%)	18 (22.4%)	10 (23.1%)	7 (18.4%)	3 (9.1%)	9 (33.3%)	7 (28.6%)	4 (7.4%)

Feeding type information before and after swallowing evaluation was missing for some patients.

**Table 4:** Comparison of videofluoroscopic swallowing study findings according to pneumonia history.

Variable	Pneumonia History (NO) (*n* = 774) *n* (%)	Pneumonia (YES) (*n* = 281) *n* (%)	*p*-Value
Aspiration—liquid	341 (44.1)	212 (75.5)	**<0.001**
Silent aspiration—liquid	186 (24.0)	137 (48.9)	**<0.001**
Aspiration—semisolid	119 (15.4)	89 (31.7)	**<0.001**
Silent aspiration—semisolid	75 (9.7)	68 (24.3)	**<0.001**
Pharyngeal residue—liquid	79 (10.2)	40 (14.2)	0.086
Pharyngeal residue—semisolid	177 (22.9)	79 (28.1)	0.127

Chi-square test note: Aspiration includes both nonsilent (PAS 6–7) and silent aspiration (PAS 8); therefore, categories are not mutually exclusive.

**Table 5:** Multivariable logistic regression analysis of VFSS findings associated with pneumonia history.

Variable	OR	95% CI	*p*-Value
Liquid Aspiration Pattern			
Nonsilent Aspiration (PAS 6–7)	2.18	1.26–3.78	0.005
Silent Aspiration (PAS 8)	3.06	1.98–4.72	<0.001
Semisolid Aspiration Pattern			
Nonsilent Aspiration (PAS 6–7)	2.32	1.32–4.08	0.003
Silent Aspiration (PAS 8)	3.71	2.15–6.40	<0.001
Pharyngeal Residue			
Liquid	1.23	0.93–1.63	0.142
Semisolid	1.57	1.09–2.26	0.015

*OR*: *odds ratio*; *CI*: *confidence interval*; PAS: Penetration–Aspiration Scale; *VFSS*: *videofluoroscopic swallowing study*.

## Data Availability

The data presented in this study are not publicly available due to ethical and privacy restrictions related to the use of hospital records but are available from the corresponding author upon reasonable request.

## Abbreviations

The following abbreviations are used in this manuscript:

VFSS	Videofluoroscopic swallowing study
PAS	Penetration–Aspiration Scale
EAT-10	Eating Assessment Tool
